# Complete Genome Sequences of Eight Human Papillomavirus Type 16 Asian American and European Variant Isolates from Cervical Biopsies and Lesions in Indian Women

**DOI:** 10.1128/genomeA.00243-16

**Published:** 2016-05-19

**Authors:** Paramita Mandal, Bornali Bhattacharjee, Shrinka Sen, Amrapali Bhattacharya, Rahul Roy Chowdhury, Nidhu Ranjan Mondal, Sharmila Sengupta

**Affiliations:** a National Institute of Biomedical Genomics, Kalyani, West Bengal, India; b Department of Gynecology, Saroj Gupta Cancer Centre and Research Institute, Kolkata, India

## Abstract

Human papillomavirus type 16 (HPV16), a member of the *Papillomaviridae* family, is the primary etiological agent of cervical cancer. Here, we report the complete genome sequences of four HPV16 Asian American variants and four European variants, isolated from cervical biopsies and scrapings in India.

## GENOME ANNOUNCEMENT

Human papillomavirus type 16 (HPV16), a member of the *Papillomaviridae* family, is phylogenetically clustered within the *Alphapapillomavirus 9* species group and is predominantly associated with cervical cancer (CaCx) (1, 2). Corresponding to geographical regions, HPV16 is classified into five variant lineages (3–5), namely, European (E), Asian (A), Asian American (AA), African-1 (Af1), and African-2 (Af2).

In India, HPV16 has been found to be the most prevalent high-risk type associated with CaCx cases (6), and, in an earlier report from our laboratory, the presence of AA variants was confirmed for the first time along with E variants using Sanger sequencing (7–9).

Here, we report the complete genome sequences of four viral isolates belonging to the AA variant lineage and four viral isolates belonging to the E variant lineage, isolated from cervical biopsies and scrapings in India.

DNA was isolated from cervical specimens using the Qiagen DNA minikit (Qiagen, Germany) according to the manufacturer’s instructions. HPV screening was carried out using broad range GP5+/GP6+ primer pairs, and the presence of HPV16 was confirmed by quantitative E6 PCR (10). Viral genomes were enriched using a 100-ng DNA template, two long-range overlapping primer sets, and an Expand Long Template PCR enzyme mix (Roche, Switzerland). The amplicons were purified, quantitated, and mixed in equimolar proportions to generate 1 µg of starting material. The pooled amplicons were subsequently sheared to low molecular weight fragments. Adapter ligation was carried out using an Ion Plus fragment library kit (Thermo Fisher Scientific, USA) and each library was labeled using Ion Xpress bar code adapters (Thermo Fisher Scientific, USA). The ligated libraries were size-selected using E‐Gel SizeSelect 2% agarose gels (Thermo Fisher Scientific, USA) and assessed on an Agilent 2100 Bioanalyzer (Agilent Technologies, Germany). The libraries were quantified using the Ion Library TaqMan quantitation kit (Thermo Fisher Scientific, USA) and the bar-coded library pools were amplified onto Ion Sphere particles by emulsion PCR. High-throughput sequencing was performed on an Ion PGM sequencer platform (Thermo Fisher Scientific, USA), and the Torrent Suite version 3.0 data processing pipeline was used to generate sequence reads. Per genome, approximately 2,300 paired-end reads with an average insert length of 200 bp were generated (~46,000 bases/genome). *De novo* assembly was carried out to form consensus sequences using the Geneious version 7.0.3 assembler (11). Whole-genome Sanger sequences, generated independently using short range primers sets (8) from each specimen, were aligned to respective consensus sequences for confirmation. Each consensus sequence was manually checked to identify variant lineages on the basis of differences in the L1 region and whole genome BLAST search. The curated genome sequences were annotated with genome annotation transfer utility software (12) using HPV16 E or AA reference sequences (NC_001526.2 and AB818689) as templates. The annotated genome sequences thus generated were further validated manually.

### Nucleotide sequence accession numbers.

Whole-genome sequences of all eight viral isolates have been deposited in GenBank using NCBI’s BankIt tool, and the accession numbers are listed in Table 1.

**TABLE 1:** List of HPV16 isolate genomes released to NCBI

Isolate ID	Accession number	Source	Genome size (bp)	Variant type
IND-T119	KU641509	Cervical biopsy	7,908	Asian American
IND-T121	KU684314	Cervical biopsy	7,908	Asian American
IND-T488	KU684311	Cervical biopsy	7,916	Asian American
IND-CMCP14	KU684315	Cervical scrape	7.909	Asian American
IND-T395	KU684313	Cervical biopsy	7,909	European
IND-T315	KU684316	Cervical biopsy	7,906	European
IND-T338	KU684317	Cervical biopsy	7,906	European
IND-JNM434	KU684312	Cervical scrape	7,906	European

## References

[1] Das BC, Gopalkrishna V, Hedau S, Katiyar S 2000 Cancer of the uterine cervix and human papillomavirus infection. Curr Sci 78:52–63.

[2] Sowjanya AP, Jain M, Poli UR, Padma S, Das M, Shah KV, Rao BN, Devi RR, Gravitt PE, Ramakrishna G 2005 Prevalence and distribution of high-risk human papilloma virus (HPV) types in invasive squamous cell carcinoma of the cervix and in normal women in Andhra Pradesh, India. BMC Infect Dis 5:116. doi:10.1186/1471-2334-5-116. 16371167 PMC1345691

[3] Cornet I, Gheit T, Iannacone MR, Vignat J, Sylla BS, Del Mistro A, Franceschi S, Tommasino M, Clifford GM 2013 HPV16 genetic variation and the development of cervical cancer worldwide. Br J Cancer 108:240–244. doi:10.1038/bjc.2012.508. 23169278 PMC3553516

[4] Ho L, Chan SY, Burk RD, Das BC, Fujinaga K, Icenogle JP, Kahn T, Kiviat N, Lancaster W, Mavromara-Nazos P 1993 The genetic drift of human papillomavirus type 16 is a means of reconstructing prehistoric viral spread and the movement of ancient human populations. J Virol 67:6413–6423. 8411343 10.1128/jvi.67.11.6413-6423.1993 PMC238076

[5] Yamada T, Wheeler CM, Halpern AL, Stewart AC, Hildesheim A, Jenison SA 1995 Human papillomavirus type 16 variant lineages in United States populations characterized by nucleotide sequence analysis of the E6, L2, and L1 coding segments. J Virol 69:7743–7753. 7494284 10.1128/jvi.69.12.7743-7753.1995 PMC189716

[6] Sharma K, Kathait A, Jain A, Kujur K, Raghuwanshi S, Bharti AC, Saklani AC, Das BC 2015 Higher prevalence of human papillomavirus infection in adolescent and young adult girls belonging to different Indian tribes with varied socio-sexual lifestyle. PLoS One 10:e0125693. doi:10.1371/journal.pone.0125693. 25954813 PMC4425665

[7] Bhattacharjee B, Sengupta S 2006 HPV16 E2 gene disruption and polymorphisms of E2 and LCR: some significant associations with cervical cancer in Indian women. Gynecol Oncol 100:372–378. doi:10.1016/j.ygyno.2005.09.016. 16246404

[8] Bhattacharjee B, Mandal NR, Roy S, Sengupta S 2008 Characterization of sequence variations within HPV16 isolates among Indian women: prediction of causal role of rare non-synonymous variations within intact isolates in cervical cancer pathogenesis. Virology 377:143–150. doi:10.1016/j.virol.2008.04.007. 18495198

[9] Cornet I, Gheit T, Franceschi S, Vignat J, Burk RD, Sylla BS, Tommasino M, Clifford GM, the IARC HPV Variant Study Group 2012 Human papillomavirus type 16 genetic variants: phylogeny and classification based on E6 and LCR. J Virol 86:6855–6861. doi:10.1128/JVI.00483-12. 22491459 PMC3393538

[10] Das D, Bhattacharjee B, Sen S, Mukhopadhyay I, Sengupta S 2010 Association of viral load with HPV16 positive cervical cancer pathogenesis: causal relevance in isolates harboring intact viral E2 gene. Virology 402:197–202. doi:10.1016/j.virol.2010.03.030. 20394955

[11] Kearse M, Moir R, Wilson A, Stones-Havas S, Cheung M, Sturrock S, Buxton S, Cooper A, Markowitz S, Duran C, Thierer T, Ashton B, Meintjes P, Drummond A 2012 Geneious Basic: an integrated and extendable desktop software platform for the organization and analysis of sequence data. Bioinformatics 28:1647–1649. doi:10.1093/bioinformatics/bts199. 22543367 PMC3371832

[12] Tcherepanov V, Ehlers A, Upton C 2006 Genome annotation transfer utility (GATU): rapid annotation of viral genomes using a closely related reference genome. BMC Genomics 7:150. doi:10.1186/1471-2164-7-150. 16772042 PMC1534038

